# High vector competence for chikungunya virus but heavily reduced locomotor activity of *Aedes albopictus* from Germany at low temperatures

**DOI:** 10.1186/s13071-024-06594-x

**Published:** 2024-12-04

**Authors:** Renke Lühken, Leif Rauhöft, Björn Pluskota, Unchana Lange, Michelle Helms, Norbert Becker, Jonas Schmidt-Chanasit, Carola Kuhn, Egbert Tannich, Stephanie Jansen, Anna Heitmann

**Affiliations:** 1 Bernhard Nocht Institute for Tropical Medicine, Hamburg, Germany; 2 Kommunale Aktionsgemeinschaft zur Bekämpfung der Schnakenplage (KABS E.V.), Speyer, Germany; 3 Institute for Dipterology (IfD), Speyer, Germany; 4 Center for Organismal Studies (COS), University of Heidelberg, Heidelberg, Germany; 5 Faculty of Mathematics, Informatics and Natural Sciences, Universität Hamburg, Hamburg, Germany; 6 German Environment Agency (UBA), Berlin, Germany

**Keywords:** Chikungunya virus, *Aedes albopictus*, Vector competence, Vector capacity, Locomotor activity, Low temperature

## Abstract

**Background:**

The incidence of human infections caused by arthropod-borne viruses, such as the chikungunya virus (CHIKV), has increased globally due to a number of factors, such as climate change and globalization. The exotic mosquito species *Aedes albopictus* is a significant vector for CHIKV, raising concerns about its transmission potential in temperate regions, including Central Europe. We have therefore investigated the vector competence of *Ae. albopictus* for CHIKV at constant and fluctuating temperatures between 15 °C and 24 °C to assess the transmission risk in Europe.

**Methods:**

*Aedes albopictus* mosquitoes were reared and artificially infected with CHIKV. Infection rates and transmission efficiencies (TEs) were determined after 14 days of incubation at constant and fluctuating (± 5 °C) mean temperatures of 15 °C, 18 °C, 21 °C and 24 °C. In addition, mosquito locomotor activity was measured under the same fluctuating temperature conditions. A risk map for CHIKV transmission in Europe was generated combining temperature data and the current distribution of *Ae. albopictus*.

**Results:**

CHIKV transmission was observed at all tested temperatures. The highest TEs were recorded at fluctuating temperatures of 18 °C (54.3%) and 21 °C (58.6%), while the lowest TE was observed at a constant temperature of 15 °C (5.6%). TEs at fluctuating temperatures of 15 °C and 24 °C were the same (32.5%). Mosquito activity showed a nocturnal unimodal activity pattern with a peak during the start of the scotophase (hour 20). The proportion of active mosquitoes per hour increased with temperature and was nearly zero at 15 °C. The risk map indicated that regions in Southern and Central Europe, including recently invaded areas north of the Alps, have temperatures theoretically allowing CHIKV transmission for at least some days per year.

**Conclusions:**

While CHIKV can be transmitted by *Ae. albopictus* at 15 °C, the activity of this mosquito is strongly decreased at this temperature, likely reducing the transmission risk. These findings emphasize the importance of considering both vector competence and mosquito activity when assessing the risk of arbovirus transmission in temperate regions. Further studies are needed to validate these laboratory findings under field conditions.

**Graphical abstract:**

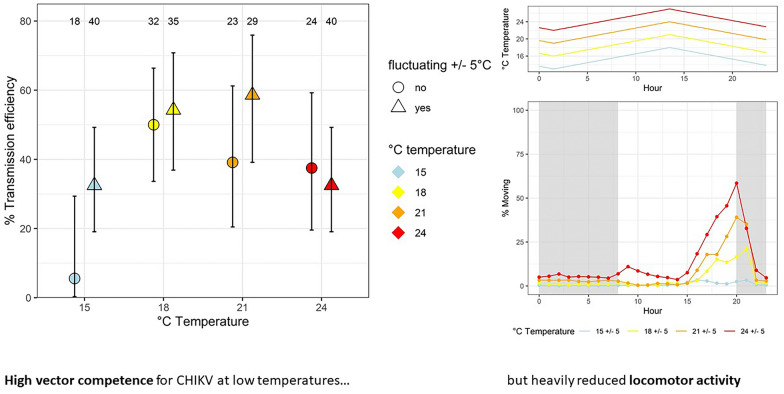

**Supplementary Information:**

The online version contains supplementary material available at 10.1186/s13071-024-06594-x.

## Background

Over the last decades, human infections caused by arthropod-borne viruses (arboviruses) have increased worldwide, as shown by significant outbreaks of dengue virus, chikungunya virus (CHIKV) and Zika virus [1]. This increase can be attributed to various factors linked to globalization and global change, such as climate warming and land-use change, which favor the spread and establishment of invasive mosquito species as well as the introduction of arboviruses to areas where they were not reported previously [2].

From the public health perspective, CHIKV is one of the most important virus species within the family *Togaviridae* (genus *Alphavirus*). This family of viruses is characterized by a single-stranded positive-sense RNA genome organized in spherical enveloped virions [3]. Human CHIKV infections are associated with fever and severe, debilitating arthralgia, both of which can become chronic for years [4]. The first CHIKV epidemics were observed in 1952–1953 at the border region between Mozambique and Tanzania [5]. Historically, CHIKV predominantly circulates on the African and Asian continent, but over the last decades it has significantly expanded its range, with huge outbreaks in India, the Indian Ocean islands and the Americas involving millions of human infections [1, 6]. Although CHIKV is not endemic in Europe, autochthonous transmission has been reported to have led to several outbreaks, with up to 300 infected individuals, caused by regular introductions of CHIKV by infected travelers [7].

CHIKV circulates in an enzootic cycle between forest-dwelling *Aedes* species and non-human primates, but an urban transmission cycle between humans and other *Aedes* species has been well established in the last decades [8]. *Aedes aegypti* is considered to be the primary vector of CHIKV. However, mutations of the East/Central/South African (ECSA) strain of CHIKV has facilitated transmission by *Ae. albopictus* 40-fold, making this latter species an important vector [9, 10]. *Aedes albopictus* has been established in Italy since the 1980s, at first spreading around the Mediterranean Sea, but also expanding its range towards Central Europe, including the establishment of populations north of the Alps in Germany [11, 12]. High vector densities in Italy, Spain and France enabled smaller and larger outbreaks of CHIKV, dengue virus and Zika virus [2, 13].

Although CHIKV is an arbovirus of global relevance, a systematic literature study only identified eight studies analyzing the CHIKV vector competence of *Ae. aegypti* and *Ae. albopictus* [14], highlighting the current lack of knowledge on the interaction between temperature and CHIKV transmission. Previous studies on the risk of CHIKV circulation in Europe under constant experimental temperature conditions demonstrated that the areas under risk might be predominantly associated with the presence and abundance of the vector species as high CHIKV transmission rates were also observed at low temperatures of 18 °C [15]. This observation led to the question of whether transmission can also be observed at even lower temperatures and if the Asian tiger mosquito still shows flight activity at these temperatures. The aim of the study reported here was to assess vector competence of *Ae. albopictus* from Germany at constant and fluctuating (± 5 °C) temperatures (15 °C, 18 °C, 21 °C, 24 °C) that better represent natural conditions. In addition, we also measured locomotor activity under the same fluctuating temperature conditions to give a more comprehensive assessment of spatial CHIKV transmission risk in Europe.

## Methods

To establish a colony of *Ae. albopictus* from Germany, eggs were collected in summer 2015 in Freiburg, Germany. Mosquitoes were reared at 26 °C and 80% humidity, under a light:dark photoperiod of 12:12 h, including 30 min twilight. Ten randomly selected specimens of the F4 generation were tested by pan-PCRs and found to be negative for flavi-, alpha- and orthobunyaviruses to exclude natural infections [16–18]. For the experiments, a total of 241 females (4–14 days old) of generation F8-F12 were analyzed. Mosquitoes were infected as described in detail by Heitmann et al. [19]. Briefly, 20 specimens were starved for 24 h in Drosophila breeding vials (Carl Roth, Karlsruhe, Germany) before feeding on an infectious blood meal for 3 h, offered in two 50-µl droplets pipetted onto the bottom of the vials. The final concentration of CHIKV (strain CNR_24/2014, supplied by the European Virus Archive goes Global Project [EVAg], ECSA-lineage, originally isolated from a human case in France, 5th passage) in the blood meal was 10^6^ plaque-forming units per milliliter (PFU/ml). Engorged females were subsequently sorted and incubated for 14 days at constant temperatures of 15 °C, 18 °C, 21 °C or 24 °C, as described in part by Heitmann et al. [15], and at fluctuating temperatures (± 5 °C) of 15 ± 5 °C, 18 ± 5 °C, 21 ± 5 °C, 24 ± 5 °C or 27 ± 5 °C, with a relative humidity of 70%. At the population level it has been modeled that > 99% of specimens exposed to temperatures ranging from 15 °C to 24 °C are expected to survive at least 14 days [19]. A salivation assay was conducted for each mosquito specimen as described in [15, 20]. In short, mosquitoes were anesthetized with CO_2_ and immobilized. To induce salivation, the mosquito's proboscis was inserted into a filter tip containing 10 µl of phosphate-buffered saline (PBS) for 30 min, following which the saliva was incubated on Vero cells for 4 days. The cells were monitored for cytopathic effects (CPE). The supernatant from the CPE-positive cells was collected, and RNA copies were detected with the RealStar Chikungunya RT-PCR Kit 2.0 (Altona Diagnostics, Hamburg, Germany) after extraction with the QIAamp Viral RNA Mini Kit (Qiagen, Hilden, Germany). All experiments were performed at least twice independently for each condition. Replicates were only included if a randomly selected, engorged female at day zero was infected with viable CHIKV, i.e. showing CPE-positive cells validated by reverse transcription PCR (RT-PCR) by the methods described above. Infection rates (number of virus-positive mosquito bodies per number of fed females) and transmission efficiencies (TEs, number of virus-positive saliva per number of fed specimens) were calculated as described by Heitmann et al. [15]. A binomial generalized linear model (GLM) was fitted with TE as the response and a Gaussian GLM was fitted with titer as response variable, both with mean temperature and the factor fluctuating/non-fluctuating as predictors.

Mosquito activity was measured using the LAM25H-3 Locomotor Activity Monitor (LAM; TriKinetics Inc, Waltham, MA, USA) with three board stack monitors at three axial positions per tube. Single females aged from 2 to 14 days were anesthetized with CO_2_ and placed into the glass tubes of the LAM. The tubes were closed on each side with ceapren plugs (Greiner Bio-One, Kremsmünster, Austria) that had been cut through the middle to enable placement of a dental Monoart cotton roll (size 1; Euronda GmbH, Altenberge, Germany) into the vial. The cotton rolls were soaked with an 8% fructose solution to ensure continuous sustenance with fructose on both sides of the vial. The tubes were inserted horizontally into the LAM, which was placed into an incubator maintained at 70% humidity and a 12:12 h light:dark photoperiod, with fluctuating temperatures of 15 ± 5 °C, 18 ± 5 °C, 21 ± 5 °C, 24 ± 5 °C, mimicking a day/night rhythm, with the peak temperature in the middle of the light period. Measurements were started on the following day. Over 3 days the number of beam-crossing events were measured in 1-min intervals with the DAMSystem3 program (v3.12.1; TriKinetics Inc). Experiments were repeated 3 times independently. For each tube and minute, mosquitoes were counted as active if one of the three monitors per tube recorded a signal. The proportion of active mosquito specimens per 10-min interval and the proportion of 10-min intervals showing mosquito activity were calculated per LAM, averaged for 60-min intervals and finally averaged over the three experiments. Spearman's rank order correlation test was applied to analyze the correlation between temperature and the mean mosquito activity for the main activity phase and the peak of activity. The correlation between mean temperature and the proportion of active specimens in the main activity phase (hour 15 to 23) and at the peak of activity (hour 20.5) was analyzed with Spearman rank correlation.

The risk map for CHIKV transmission in Europe was estimated by identifying areas in Europe presenting the temperature data used in the vector competence studies and specifically presenting the areas already colonized by *Ae. albopictus*. Daily mean temperature data (European re-analysis and observations for monitoring; E-OBS, v29.0e) were obtained from http://www.ecad.eu [21]. These E-OBS data, available on a 0.1° regular latitude–longitude grid, were extracted for a 5-year period from 2019 to 2023. For each grid cell, the number of days per year with the preceding 14 days having a mean daily temperature of ≥ 15 °C, ≥ 18 °C, ≥ 21 °C and ≥ 24 °C were calculated. The annual values were then averaged over the 5-year period. The distribution data of *Ae. albopictus* at the regional administrative level (NUTS3) as of 28 August 2023 were obtained from the European Centre for Disease Prevention and Control [22].

All calculations and visualizations were conducted with the program R [23], using the following packages: dplyr [24], tidyr [25], ggplot2 [26], stringr [27], plyr [28], lubridate [29], terra [30], tidyterra [31], RcppRoll [32], geodata [33] and ggpubr [34].

## Results

**Fig. 1:**
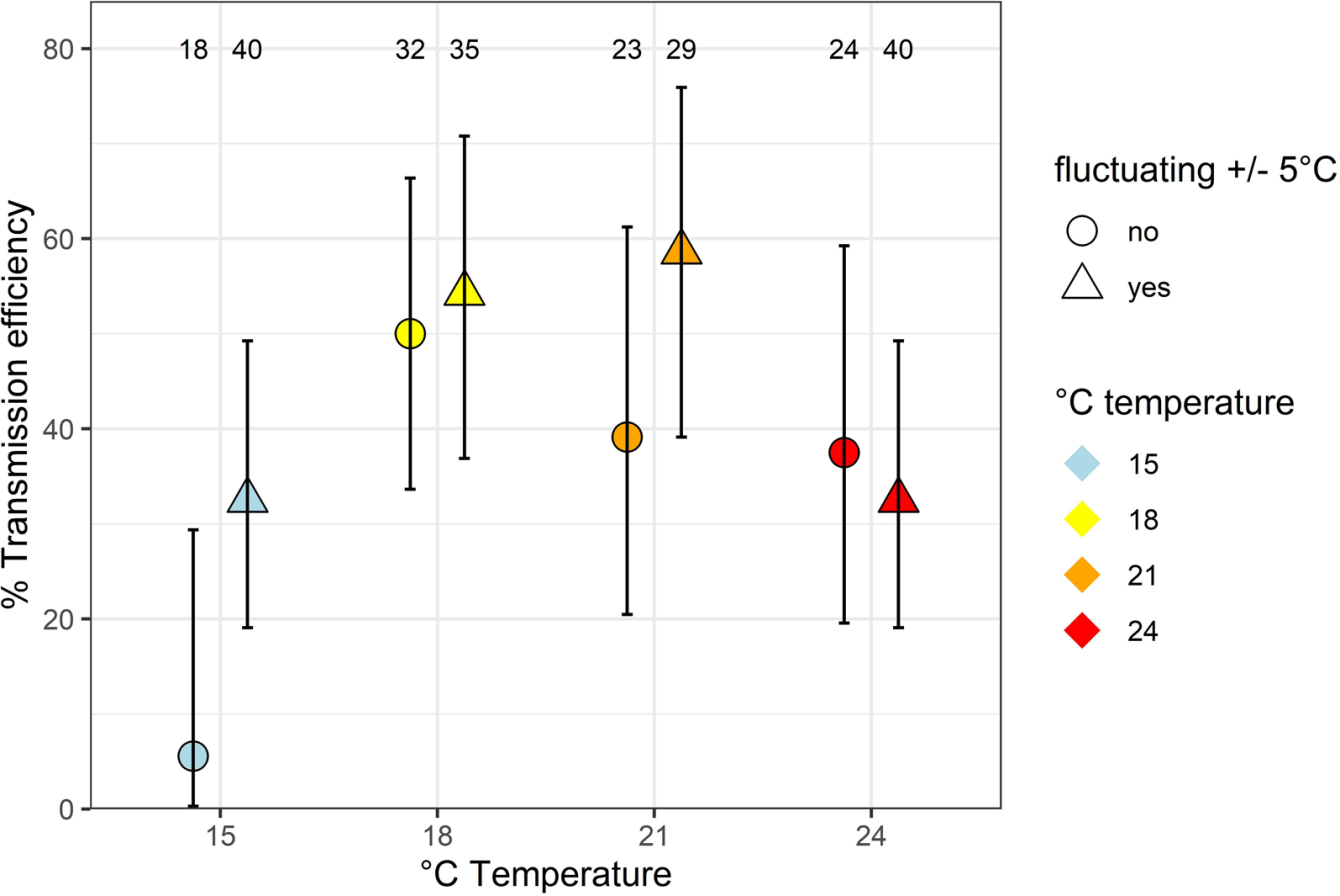
Mean transmission efficiency with 95% confidence intervals (whiskers) of *Aedes albopictus* from southern Germany under four different fluctuating (± 5 °C) and not fluctuating (constant) temperature conditions. Numbers at the top of the graph indicate the number of specimens analyzed

Infection rates of 100% were observed for all experiments (Table 1). Virus transmission was observed at all temperatures, both under the constant or fluctuating temperature conditions (Table 1; Fig. 1). Highest TEs of > 50% were observed for the fluctuating 18 °C (54.3%) and 21 °C (58.6%) temperature conditions, while for the same mean temperatures TEs were lower for constant conditions (18 °C: 50.0%; 21 °C: 39.1%). TEs at 24 °C (constant temperature: 37.5%, fluctuating temperature: 32.5%) and 15 °C (fluctuating temperature: 32.5%) were similar, while the lowest TE was observed for the 15 °C constant temperature condition (5.6%). However, there was no statistically significant impact of temperature (GLM, *df* = 1, deviance = 0.06, *P* = 0.89) whether the temperature was constant or fluctuating (GLM, *df* = 1, deviance = 0.11, *P* = 0.74) or the interaction of both variables (GLM, *df* = 1, deviance = 0.07, *P* = 0.78). The mean log10 CHIKV RNA copies per mosquito specimen ranged from 7.7 to 11.4 (Additional file 1: Figure S1), but again no statistical differences were found between the different temperature conditions (GLM, *df* = 1, *P* > 0.05).

**Table 1:** Vector competence of *Aedes albopictus* females from Germany for chikungunya virus

Temperature condition (°C)	Number of mosquitoes tested	Positive saliva	Infection rate [%]	Transmission efficiency [%]
15	18	1	100 [18/18]	5.56 [1/18]
15 ± 5	40	13	100 [40/40]	32.5 [13/40]
18	32	16	100 [32/32]	50 [16/32]
18 ± 5	35	19	100 [35/35]	54.3 [19/35]
21	23	9	100 [23/23]	39.1 [9/23]
21 ± 5	29	17	100 [29/29]	58.6 [17/29]

**Fig. 2:**
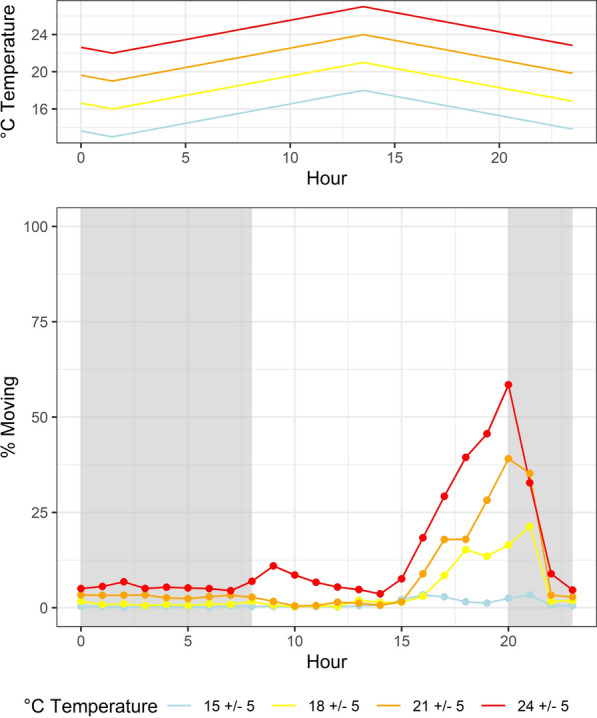
Average locomotor activity of *Aedes albopictus* from southern Germany across at least three independent experiments as percentage of specimens moving (lower panel) under four different fluctuating temperatures (upper panel)

*Aedes albopictus* females showed a nocturnal unimodal activity pattern (Fig. 2). Mosquito activity for all temperatures started to increase in the middle of the photophase (hour 15) and peaked during the start of the scotophase (hour 20) followed by a rapid decline over the following 3 h. The mean proportion of specimens active per 10-min interval between hour 15 and hour 23 significantly increased with increasing temperature from 2.5% at 15 °C to 29.7% at 24 °C (ρ = 0.99, *p*-value = 0.002). In the same way, the proportion significantly increased for the activity peak with increasing temperature (hour 20), showing an average of 2.5% at 15 °C and 58.5% at 24 °C (ρ = 0.99, *p*-value = 0.004), as did the proportion of 10-min intervals per hour with mosquito activity, increasing from 1.0% at 15 °C to 14.2% at 24 °C.

**Fig. 3:**
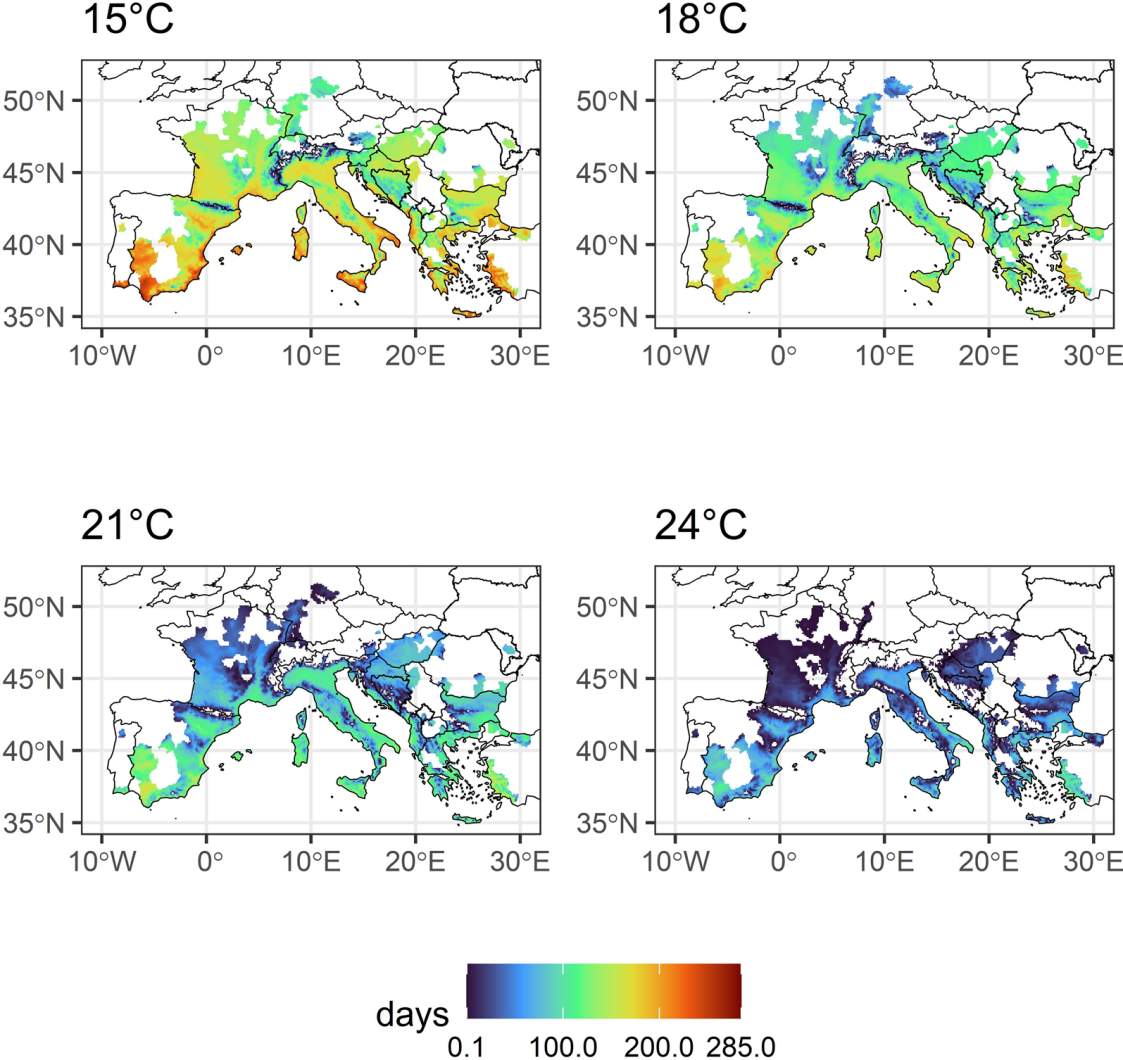
Average number of days per year with the preceding 14 days having a mean daily temperature ≥ 15 °C, ≥ 18 °C, ≥ 21 °C and ≥ 24 °C, respectively, for the period 2018–2023 for the current distribution of *Aedes albopictus* in Europe

The average number of days per raster cell (2019–2023) with the preceding 14 days having a mean daily temperature ≥ 15 °C, ≥ 18 °C, ≥ 21 °C and ≥ 24 °C, respectively, showed a clear gradient from the Mediterranean to Central Europe (Fig. 3). Regarding the current distribution of *Ae. albopictus* in Europe, we documented at least a few suitable days for all four temperatures, including the recently invaded areas north of the Alps. However, areas with historic CHIKV outbreaks in southern France or at Italy’s east coast showed relatively higher numbers of days compared to more recently invaded areas, also for the temperatures 21 °C and 24 °C.

## Discussion

Previous studies demonstrated that CHIKV can be transmitted at constant temperatures as low as 18 °C, which has led to the interpretation that the risk of CHIKV transmission in Europe is predominantly limited by the distribution and abundance of *Ae. albopictus* as a highly susceptible vector [15]. These results are confirmed in the experiments conducted in the present study, which were repeated under fluctuating temperature conditions and also included an average temperature of 15 °C. CHIKV transmission was observed at all temperatures ranging from 15 °C to 24 °C at both constant and fluctuating (± 5 °C) temperatures. Several studies have confirmed that CHIKV can be experimentally transmitted by *Ae. albopictus* at relatively low incubation temperatures compared to other arboviruses, such as dengue virus or Zika virus, with CHIKV transmission detected for constant temperature conditions of 22 °C [35], 20 °C [36, 37] and 18 °C [15, 38] or for a fluctuating temperature condition of 20 ± 6 °C [36].

TEs were higher at 18 °C and 21 °C than at 24 °C, while the TE was similar at the fluctuating temperatures of 15 °C and 24 °C. Although our results are not statistically significant and previous results from different studies indicate that increasing temperature correlates with increasing vector competence [14], our findings support previous observations of a positive impact of low temperatures on CHIKV vector competence for *Ae. albopictus* laboratory colonies from Germany, Italy and West Africa [15, 39]. The underlying theory is that some mosquito species may exhibit a reduced capacity to control viral infections at low temperatures, likely due to a temperature-dependent deficiency in their antiviral immune response [39]. Specifically, RNA silencing is inhibited in mosquitoes exposed to cooler conditions. For example, *Ae. aegypti* mosquitoes reared at lower temperatures show impairments in the RNA interference (RNAi) pathway, which is crucial for controlling viral infections. While it is well established that RNAi impairments occur downstream of the initial dicing step, the precise relationship between temperature and virus replication requires further investigation.

Fluctuating temperatures provide a more realistic simulation of the real-world temperatures observed in the field [40]. However, surprisingly few studies have systematically compared vector competence under constant and fluctuating temperatures. The range of fluctuation relative to the mean temperature around which the fluctuation takes place is considered to have a significant impact on vector competence [40]. For example, large temperature fluctuations at high temperatures was found to result in reduced vector competence of *Ae. aegypti* for dengue virus, while the fluctuation range at low temperatures increased vector competence [40, 41]. In contrast, fluctuating temperatures around 28 °C with a range of 11 °C and 15 °C did not affect transmission rates for *Culex tarsalis* and *Culex quinquefasciatus* for West Nile virus [42]. In comparison to constant temperatures, in our study mean TEs at fluctuating temperatures were higher at 15 °C, 18 °C and 21 °C, with the reverse true for 24 °C, but the differences were not statistically significant, as also demonstrated for *Ae. albopictus* from Florida [43].

Average temperatures of at least 15 °C over 14 days are very common in Europe, including areas north of the Alps, which have been recently by the Asian tiger mosquito [12]. When only temperature-dependent vector competence is considered alone, we found a high CHIKV transmission risk for the entire current distribution of *Ae. albopictus* in Europe. However, although we observed relatively high TEs for CHIKV at fluctuating temperatures of 15 °C, mosquito activity nearly dropped to zero at this temperature. Thus, although CHIKV can develop within the mosquito at very low temperatures, this does not mean that *Ae. albopictus* can actually transmit the virus at these temperatures in the field. The authors of different studies concluded that a mean daily temperature of 13 °C is the threshold allowing *Ae. albopictus* activity in the field [44]. Thus, the areas with a substantial number of days per year allowing CHIKV transmission and sufficient activity are probably restricted to Southern Europe.

Linking laboratory data to the actual situation in the field must be interpreted with caution. Mosquitoes are known to actively visit microclimates, such as resting sites, which do not necessarily represent the available large-scale temperature data based on weather stations [44–47]. Therefore, it is unclear how the presented laboratory data are transferable to field conditions. Furthermore, several general conditions of the experiments need to be considered when interpreting the results. We used female mosquitoes that varied greatly in age from 2 to 14 days for the vector competence and activity experiments; however, vector competence may be reduced in older specimens, as observed with malaria parasites [48]. Furthermore, the activity of mosquitoes was recorded in the absence of stimuli, such as host presence or other factors known to affect vector activity, including infection status [49]. Although only engorged specimens were selected after feeding on an infectious blood meal, the CHIKV body titer per female at day zero was unknown. However, it is well known that the size of the female mosquito also has an impact on the size of the blood meal [50], and the CHIKV titer might also be affected by the form of administration of the blood meals as droplets at room temperature. In addition, the mosquito colony used in the present study is relatively old (F8-F12 generation), and it is therefore possible that the results do not reflect the transmission phenotype of field mosquitoes [51]. Finally, a larger sample size might result in statistically significant differences, although we assume that epidemiologically relevant differences in vector competence should be seen within the sample size of approximately 30 specimens per temperature condition.

## Conclusions

Vector capacity is a multifactorial integral summarizing the different parameters that enable arthropods to be vectors of pathogens [52]. For example, for CHIKV, it has been demonstrated that the vector competence of *Ae. albopictus* is affected by a combination of mosquito population, viral strain and temperature [37]. Our study confirmed that vector competence is not the only variable which should be considered when estimating the temperature-dependent transmission risk. In particular, when evaluating the vector competence at the lower range of temperatures, information on vector activity should be also included in the interpretation of results.

## Supplementary Information

**Additional file 1: Figure S1**. Mean CHIKV RNA copies per *Aedes albopictus* specimen from southern Germany with 95% confidence intervals under four different fluctuating and not fluctuating temperatures. Numbers at top of figure indicate the number of specimens analyzed.

## Data Availability

No datasets were generated or analyzed during the current study.
