# Supplementary figures and images for: High vector competence for chikungunya virus but heavily reduced locomotor activity of Aedes albopictus from Germany at low temperatures
Source: Parasit Vectors. 2024 Dec 4;17:502. doi: 10.1186/s13071-024-06594-x (PMC11619113; doi:10.1186/s13071-024-06594-x)

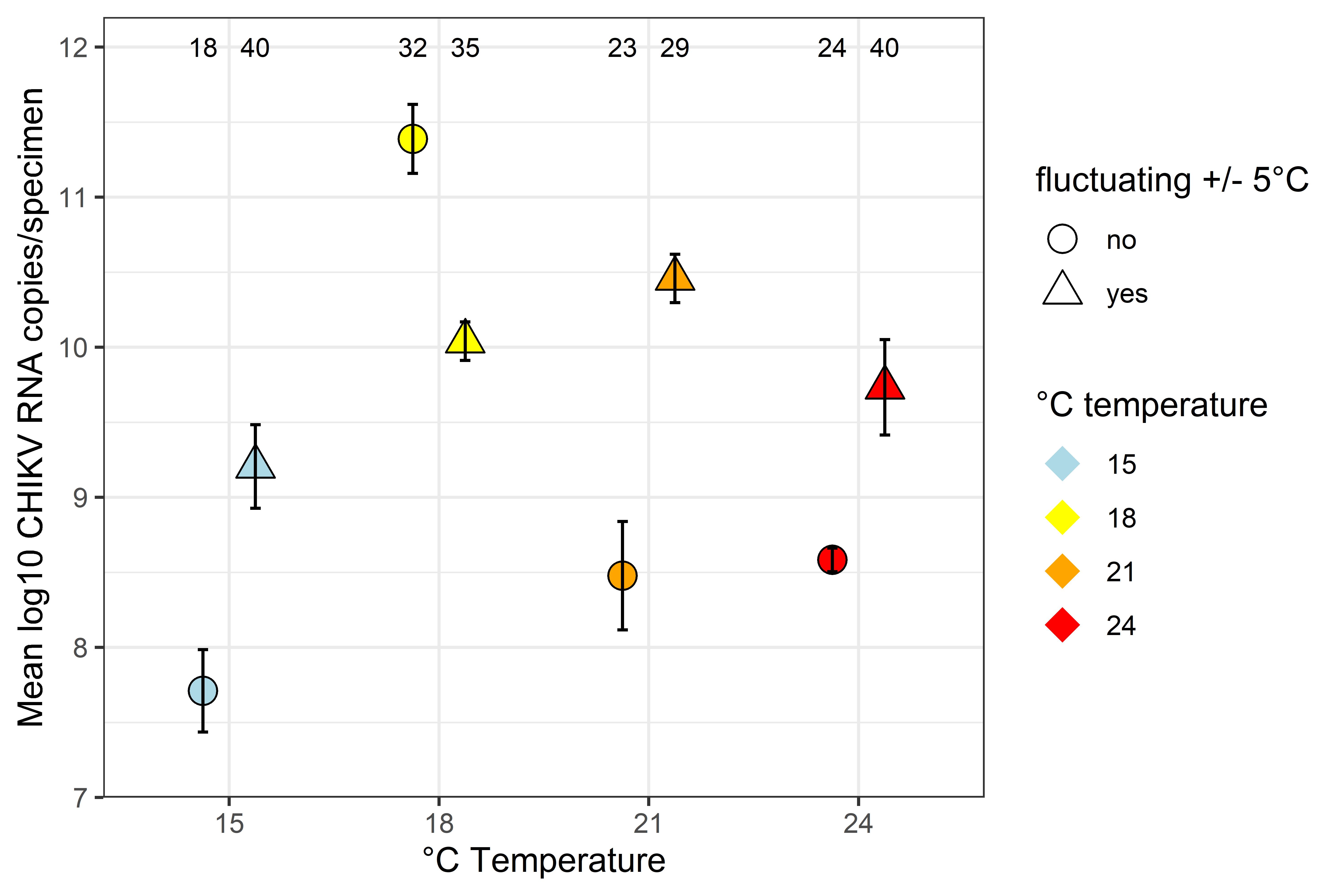

Supplement: Supplementary file 1 — Additional file 1: Figure S1. Mean CHIKV RNA copies per Aedes albopictus specimen from southern Germany with 95% confidence intervals under four different fluctuating and not fluctuating temperatures. Numbers at top of figure indicate the number of specimens analyzed. [file 13071_2024_6594_MOESM1_ESM.jpg]
